# Supplementary material for: CryoEM and computational modeling structural insights into the pH regulator NBCn1
Source: Nat Commun. 2025 Nov 11;16:9932. doi: 10.1038/s41467-025-64868-z (PMC12606367; doi:10.1038/s41467-025-64868-z)
Supplement: Supplementary file 1 — Supplementary Information [file 41467_2025_64868_MOESM1_ESM.pdf]

Supplementary information

CryoEM and Computational Modeling Structural Insights into the pH Regulator NBCn1

Wang et al.

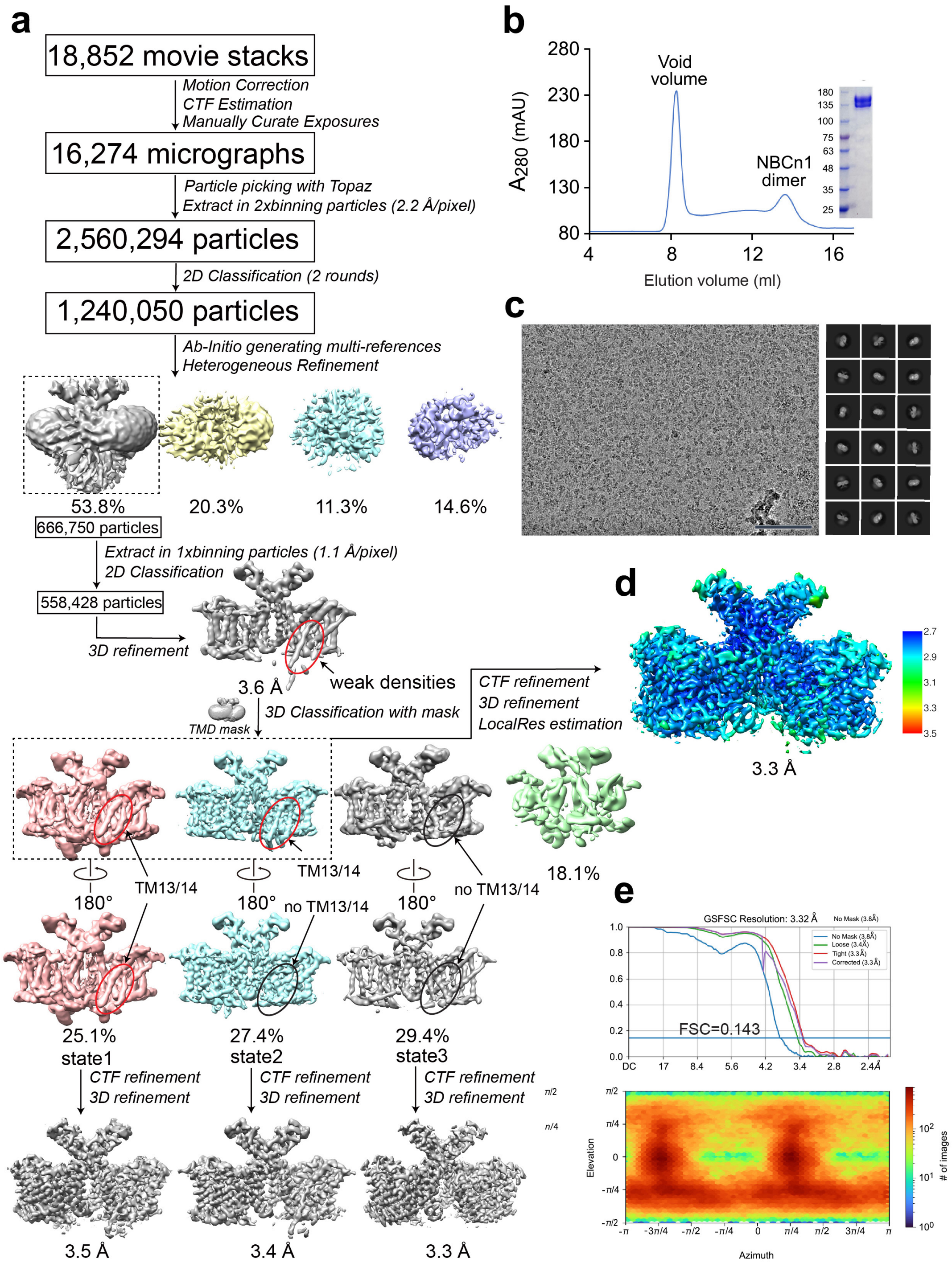

Supplementary Fig. S1. CryoEM data processing of NBCn1. a CryoEM data processing workflow for human NBCn1. **b** Gel-filtration and SDS-PAGE of NBCn1. **c** A representative cryoEM micrograph of NBCn1 (bar = 120 nm) and 2D average classes. **d** Local resolution estimation of the consensus map of NBCn1 by Resmap[Supp ref 1]. **e** Fourier shell correlation (FSC) plots and orientation distribution of particles for the final cryoEM map of NBCn1. Resolution was estimated at FSC = 0.143 (gold-standard).

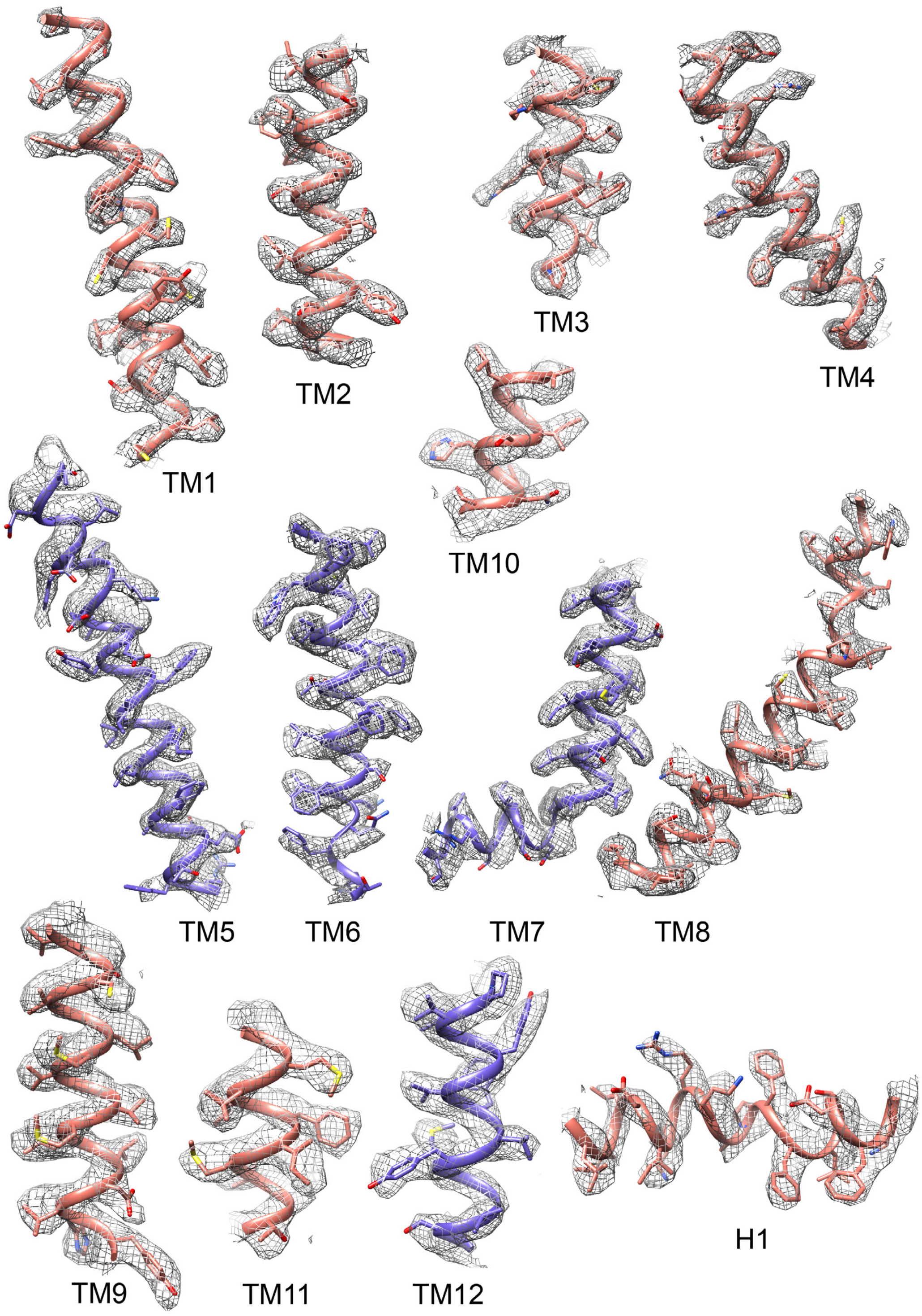

Supplementary Fig. S2. Representative cryoEM density maps of NBCn1 TMs1-14 and H1 superposed with the atomic models.

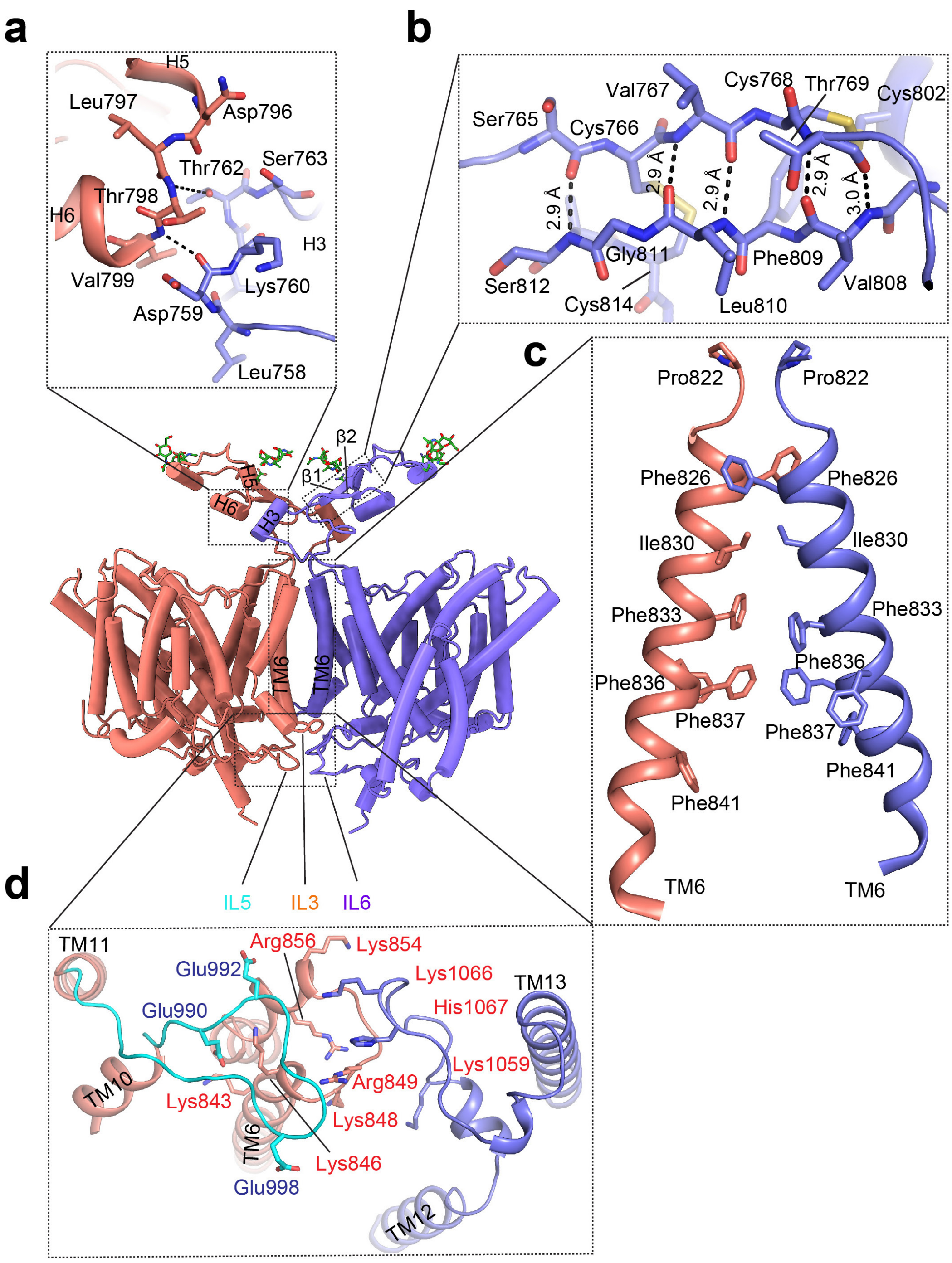

Supplementary Fig. S3. NBCn1 dimerization and intramolecular interactions. a The dimeric interactions between the H5-loop-H6 and neighboring H3 of the opposite subunit. **b** Hydrogen bonds between the anti-parallel β-strands and two intramolecular disulfide bonds, which stabilizes the EL3 assembly. **c** Hydrophobic interactions between TM6s of the two subunits. **d** Electrostatic interactions between IL5 (blue) and IL3 (salmon) and IL6 (medium purple).

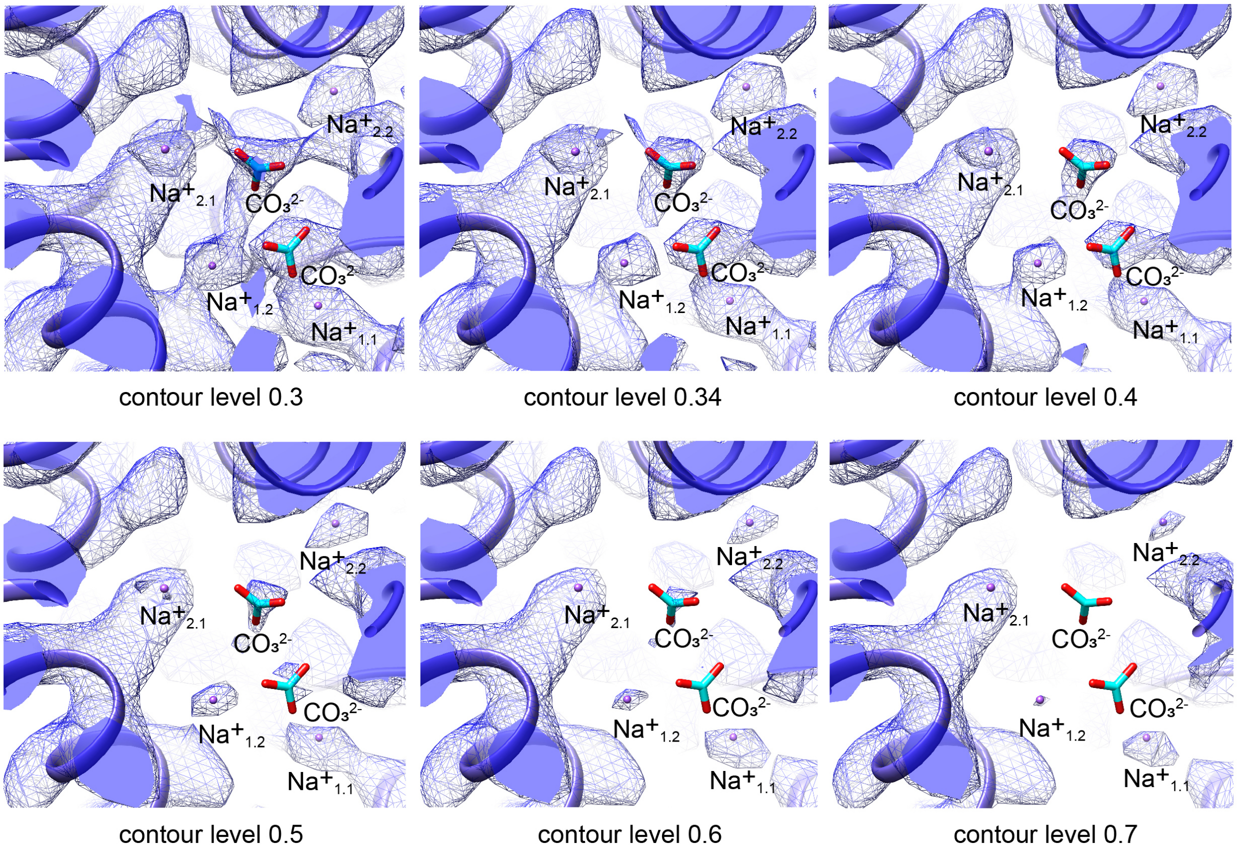

Supplementary Fig. S4. S1^{cryoEM}(OF) and S2^{cryoEM}(OF) ion binding sites in the cryoEM map shown at different contour levels. At both sites, the two densities persist across a wide range of contour levels and maintain a strong signal intensity compared to the density of putative CO_3^{2-} consistent with 2 Na^+ ions.

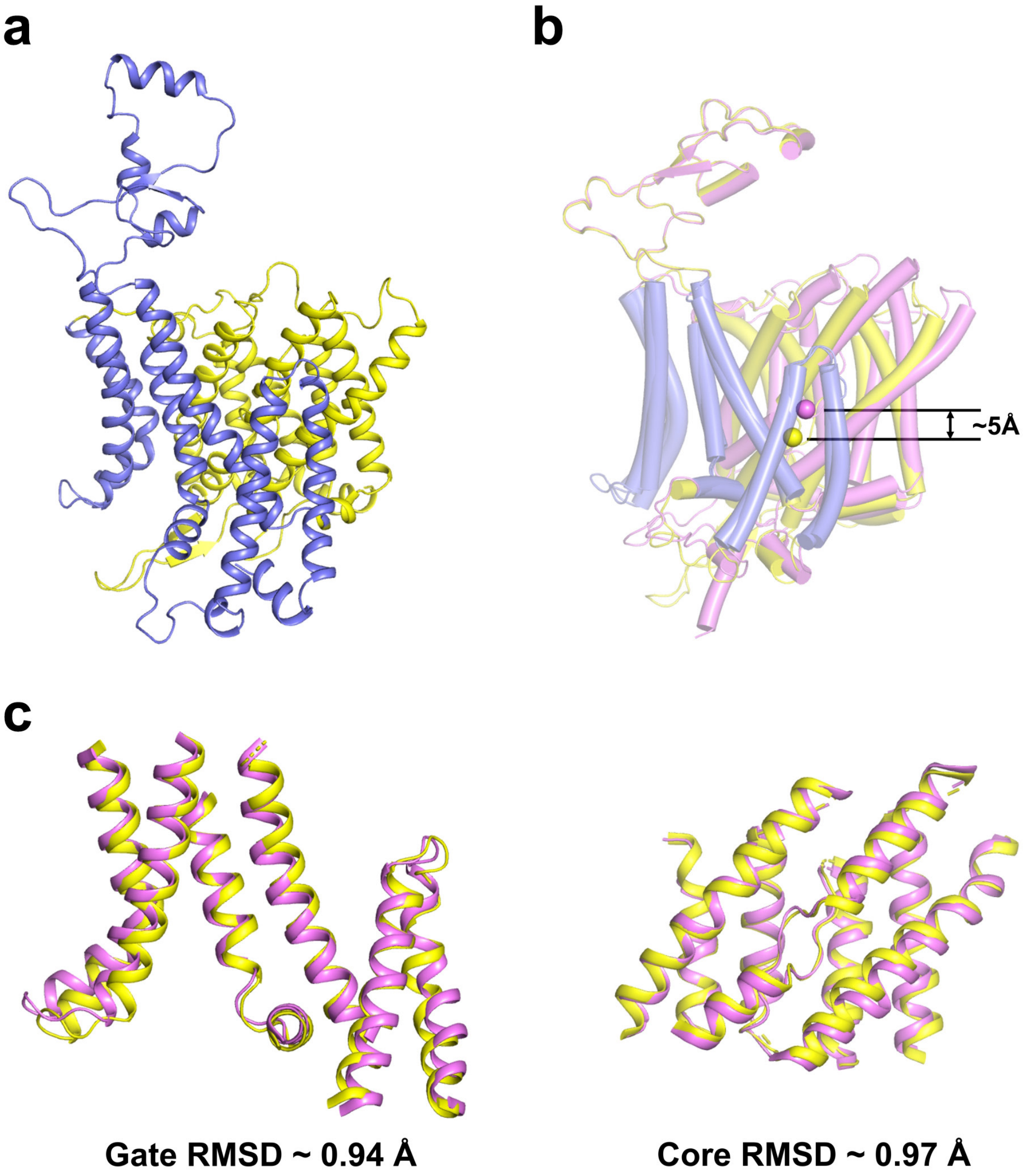

Supplementary Fig. S5. Comparison of OF and IF NBCn1 conformational states. a IF homology model of NBCn1: gate (purple helices), core (yellow helices). **b** Overlap of OF and IF states, aligned by the gate (purple cylinders). The OF and IF core domains are shown as pink and yellow cylinders, respectively. The centers of mass of the core residues from $S1^{MD}$(OF/IF) sites (Fig. 3d) are shown as pink and yellow spheres. The downward motion of ~5 Å is indicated as well. **c** (Left) Overlap of the gate domains of the OF (pink helices) and IF (yellow helices) states. (Right) Overlap of the core domains of the OF (pink helices) and IF (yellow helices) states. The corresponding RMSD values are listed.

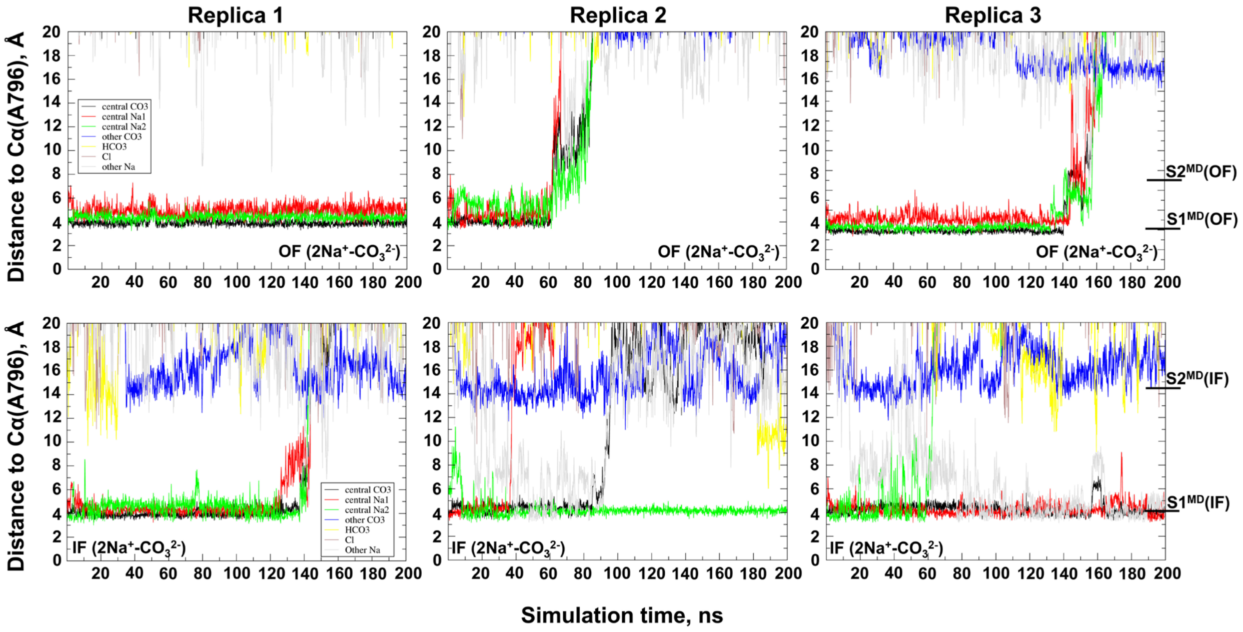

Supplementary Fig. S6. Ion time series from 200 ns MD simulations of OF and IF NBCn1 with a 2Na$^+$-CO_3^{2-} ion load. The Cα atom of residue A796 from the beginning of TM10 was selected as a proxy to the protein center and distances of various ions to the Cα atom were plotted for each of the 200 ns MD trajectories. The color coding of the lines is as follows: black (CO_3^{2-} ions bound at the central site S1 at the beginning of the MD simulations), red (a Na$^+$ bound together with the CO_3^{2-} ion at site S1 at the beginning of the MD simulations), green (the second Na$^+$ bound together with the CO_3^{2-} at site S1 at the beginning of the MD simulations, blue (CO_3^{2-} from the simulation solution), yellow (HCO_3^- from the simulation solution), brown (Cl^- from the simulation solution), grey (Na$^+$ from the simulation solution). The position of the S1/2^{MD}(OF/IF) are indicated as well.

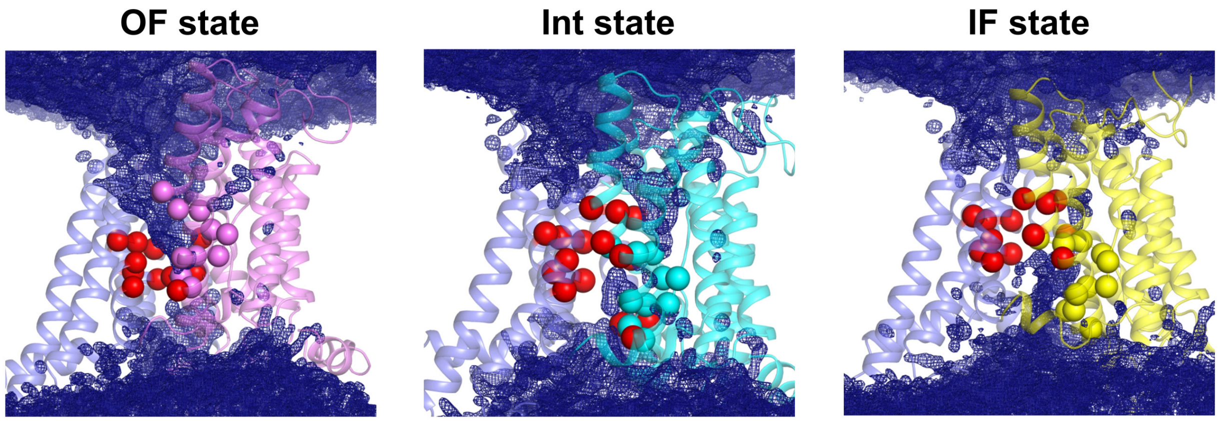

Supplementary Fig. S7. Water density maps of the NBCn1 OF, Int, and IF conformational states. Water (dark blue mesh), gate domains (purple helices), core domains (pink, cyan, and yellow helices in the OF, Int, and IF state, respectively), and Cα atoms of the $S1^{MD}$(OF/Int/IF) residues as spheres in pink, cyan, and yellow.

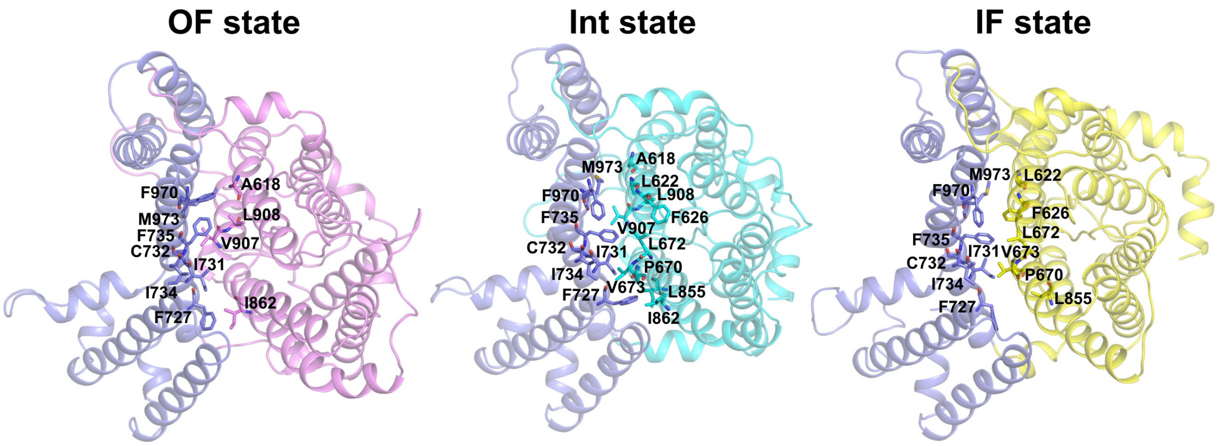

Supplementary Fig. S8. Hydrophobic residues involved in the contact interfaces during the OF↔IF transition. Gate domain (purple), OF core domain (pink), Int core domain (cyan) and IF core domain (yellow).

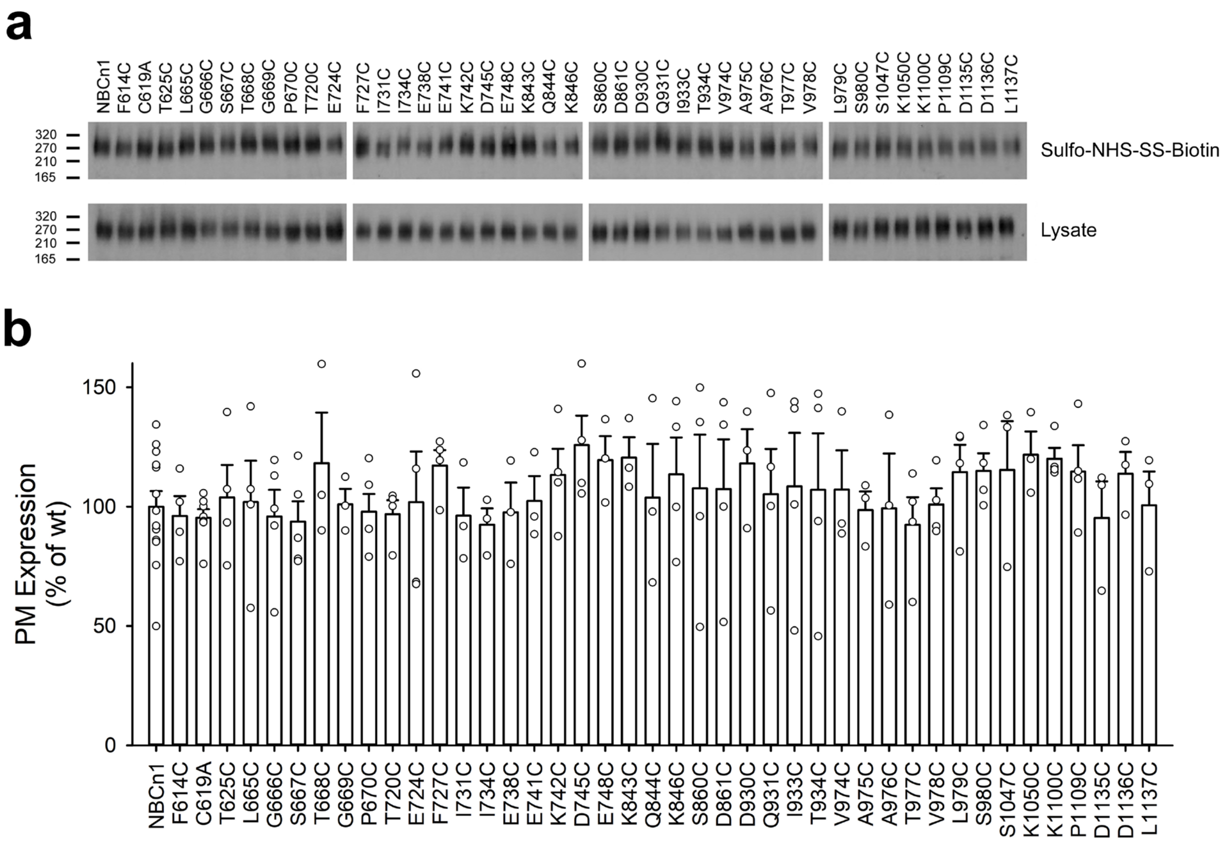

Supplementary Fig. S9. Plasma membrane wt and mutant NBCn1 protein expression. a Representative immunoblots of cell-surface and cell-lysate expression of wt and mutant NBCn1 proteins. The positions of molecular weight size markers (kDa) are shown on the left. Blot splicing is indicated with a vertical white line. Source data are provided as a Source Data file. **b** Densitometry analysis of cell-surface expression (depicted as percent of wt-NBCn1). One-way ANOVA was used to compare multiple study group means with wt-NBCn1. Mutant NBCn1 data was not statistically different from wt-NBCn1. Results are depicted as mean ± SEM (n = 3 -13 biologically independent experiments). NBCn1 wt (n = 13 biologically independent experiments) and single cysteine functional mutant data: F614C (n = 4, p = 1.0000); C619A (n = 7, p = 1.0000); T625C (n = 4, p = 1.0000); L665C (n =4, p = 1.0000); G666C (n = 5, p = 1.0000); S667C (n =5, p = 1.0000); T668C (n = 3, p = 1.0000); G669C (n = 3, p = 1.0000); P670C (n = 5, p = 1.0000); T720C (n = 4, p = 1.0000); E724C (n = 4, p = 1.0000); F727C (n = 4, p = 0.9999); I731C (n = 3, p = 1.0000); I734C (n = 3, p = 1.0000); E738C (n =3, p = 1.0000); E741C (n = 3, p = 1.0000); K742C (n = 4, p = 1.0000); D745C (n = 4, p = 0.9432); E748C (n = 3, p =0.9999); K843C (n = 3, p = 0.9997); Q844C (n = 3, p = 1.0000); K846C (n = 4, p = 1.0000); S860C (n = 4, p = 1.0000); D861C (n = 4, p = 1.0000); D930C (n = 3, p = 1.0000); Q931C (n = 4, p = 1.0000); I933C (n = 4,

p = 1.0000); T934C (n = 4, p = 1.0000); V974C (n = 3, p = 1.0000); A975C (n = 3, p = 1.0000); A976C (n = 3, p = 1.0000); T977C (n = 4, p = 1.0000); V978C (n = 4, p = 1.0000); L979C (n = 4, p = 1.0000); S980C (n = 4, p = 1.0000); S1047C (n = 3, p = 1.0000); K1050C (n = 3, p = 0.9991); K1100C (n = 4, p = 0.9983); P1109C (n = 4, p = 1.0000); D1135C (n = 3, p = 1.0000); D1136C (n = 3, p = 1.0000); and L1137C (n = 3, p = 1.0000). Open circles represent individual data points. Source data are provided as a Source Data file. Residues are numbered based on NBCn1-A for structural comparison.

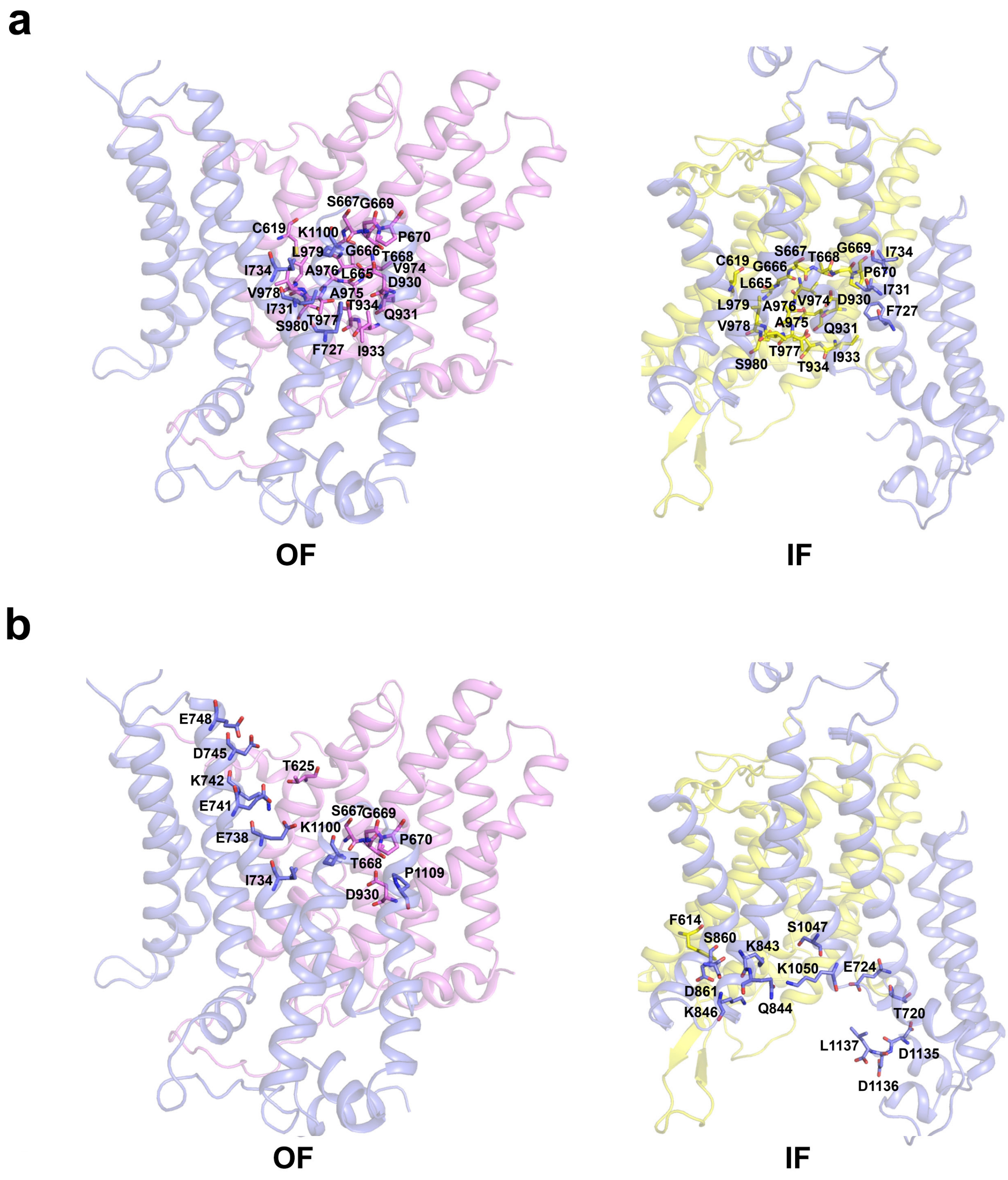

Supplementary Fig. S10. NBCn1 residues from the S1 and S2 sites and permeation pathways analyzed functionally. a Residues in the vicinity of the S1 site. **b** Residues in the vicinity of the permeation pathway and the S2 site. Gate domain (purple), OF core domain (pink) and IF core domain (yellow).

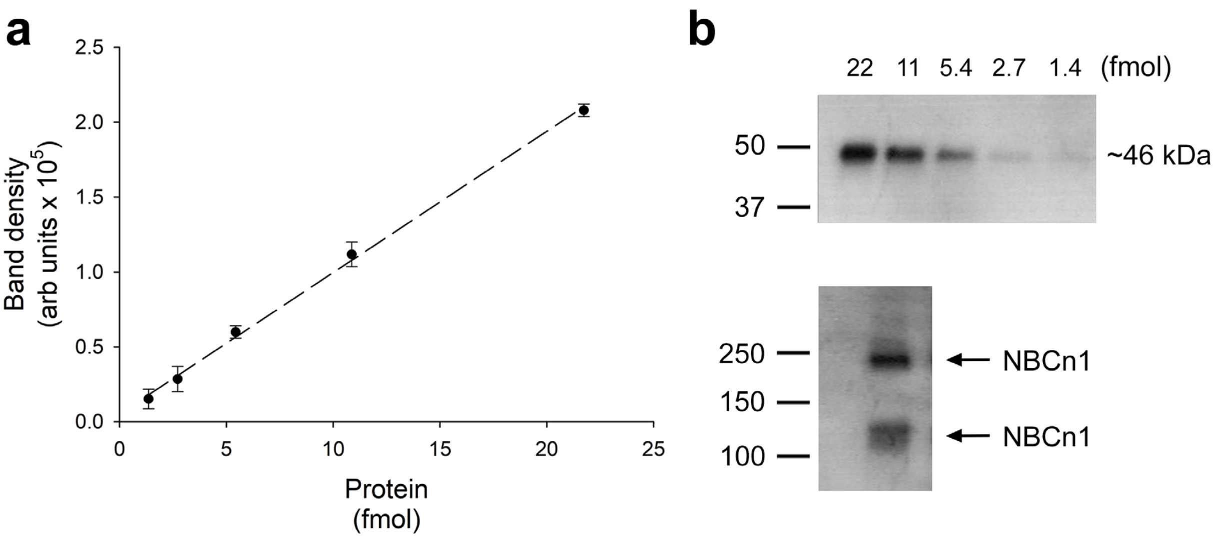

Supplementary Fig. S11. Plasma membrane monomer number quantitation. a V5 peptide standards calibration curve. The data represents the mean ± SEM. The dotted line is a linear fit to the data. **b (Top)** Immunoblot showing different amounts of the V5 peptide standards. **b (Bottom)** Immunoblot of biotinylated V5-tagged NBCn1 loaded onto the same 4-15% polyacrylamide gels as the V5 peptide standards. The upper band represents dimers and the lower band monomers. The intensity of both monomeric and dimeric bands was measured and combined to determine the total number of plasma membrane monomers. Eight separate experiments were analyzed.

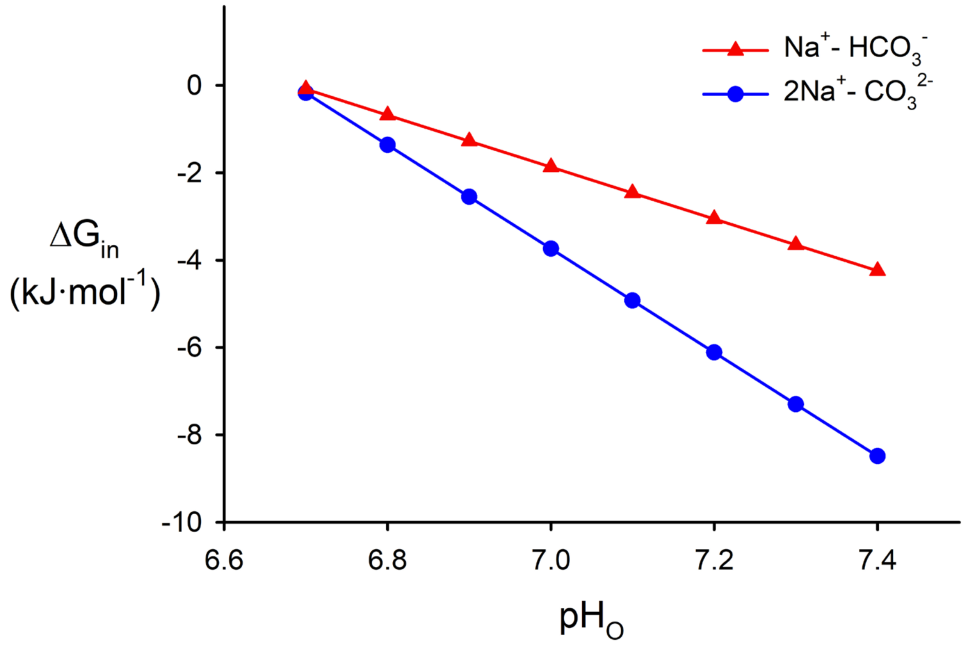

Supplementary Fig. S12. Comparison of the inwardly directed thermodynamic driving force (ΔG_{in}) for two potential NBCn1 transport modes: $2Na^{+}$–CO_3^{2-} (blue) versus Na^{+}–HCO_3^{-} (red), mediating cellular base influx (CO_3^{2-} or HCO_3^{-}) in breast tumors. Intracellular (i) and extracellular (o) ion concentrations representative of breast cancer tumor microenvironments were used[3,15,31,48-50]: $[Na^{+}_{o}]$ = 140 mM, $[Na^{+}_{i}]$ = 27 mM, pH_i = 7.4, $[CO_3^{2-}{}_{i}]$ = 0.0367 mM, and $[HCO_3^{-}{}_{i}]$ = 24.3 mM. Under these conditions, the inward chemical driving force (ΔG_{chem}) denoted ΔG_{in} (to indicate the inward transport direction) is consistently more negative, reflecting a stronger driving force for the $2Na^{+}$-CO_3^{2-} transport mode compared with Na^{+}-HCO_3^{-} transport across all pH_i (7.4) – pH_o gradients until pH_o is ~6.7.

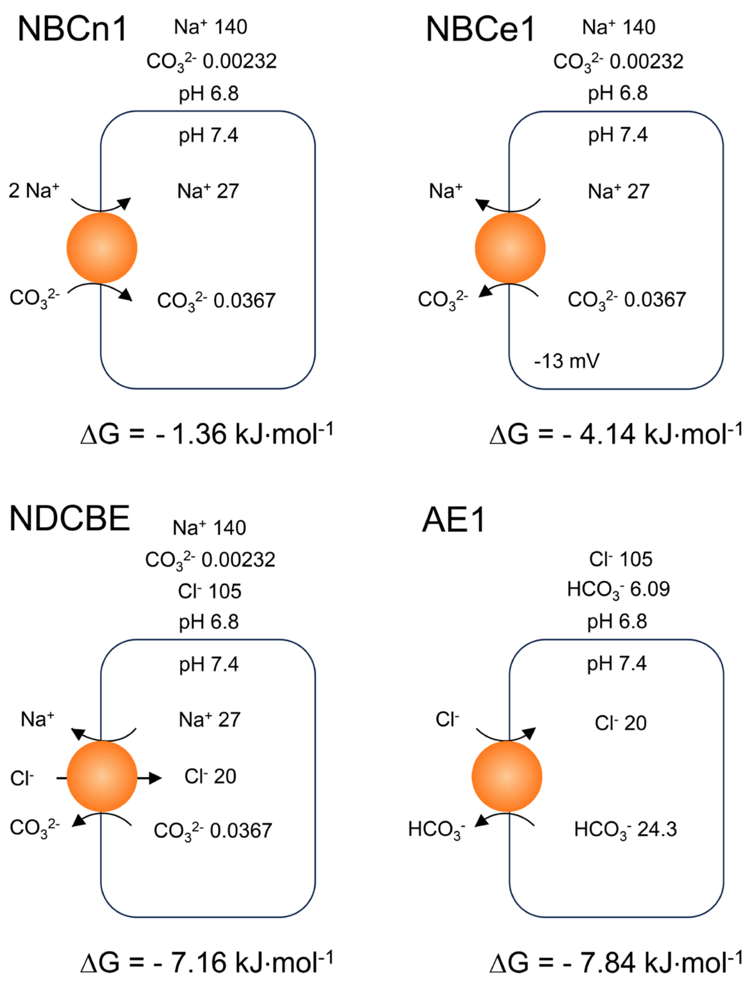

Supplementary Fig. S13. Thermodynamic driving forces (ΔG) for various SLC4 transporters mediating CO_3^{2-} or HCO_3^- flux. Examples of SLC4 transporters and corresponding transport modes that breast cancer tumors could theoretically utilize to acidify the extracellular environment and increase pH_i include NBCn1 ($2Na^+$-CO_3^{2-}), NBCe1 (Na^+-CO_3^{2-}), NDCBE (Na^+-CO_3^{2-}/Cl^-) and AE1 (Cl^-/HCO_3^-). The calculations assumed a transcellular pH gradient of 0.6 (extracellular pH 6.8; intracellular pH 7.4). The intracellular (i) and extracellular (o) ion concentrations (in mM) and cell membrane potential typical of breast tumors were used[3,15,31,48-50,Supp ref 2]. Data for $[Cl^-_i]$ are not available in breast tumors (estimated from typical values in other mammalian cells). For the electroneutral transporters (NBCn1, NDCBE and AE1), the chemical driving force (ΔG_{chem}) was calculated to determine the direction of transport. In contrast, NBCe1 is electrogenic and driven by both a chemical (ΔG_{chem}) and an electrical driving force (ΔG_{elec}; membrane potential). The calculated ΔG values for each transporter support the base transport direction (CO_3^{2-} or HCO_3^-) depicted. As shown, only in the case of NBCn1 do the thermodynamic driving forces favor tumor cellular base influx.

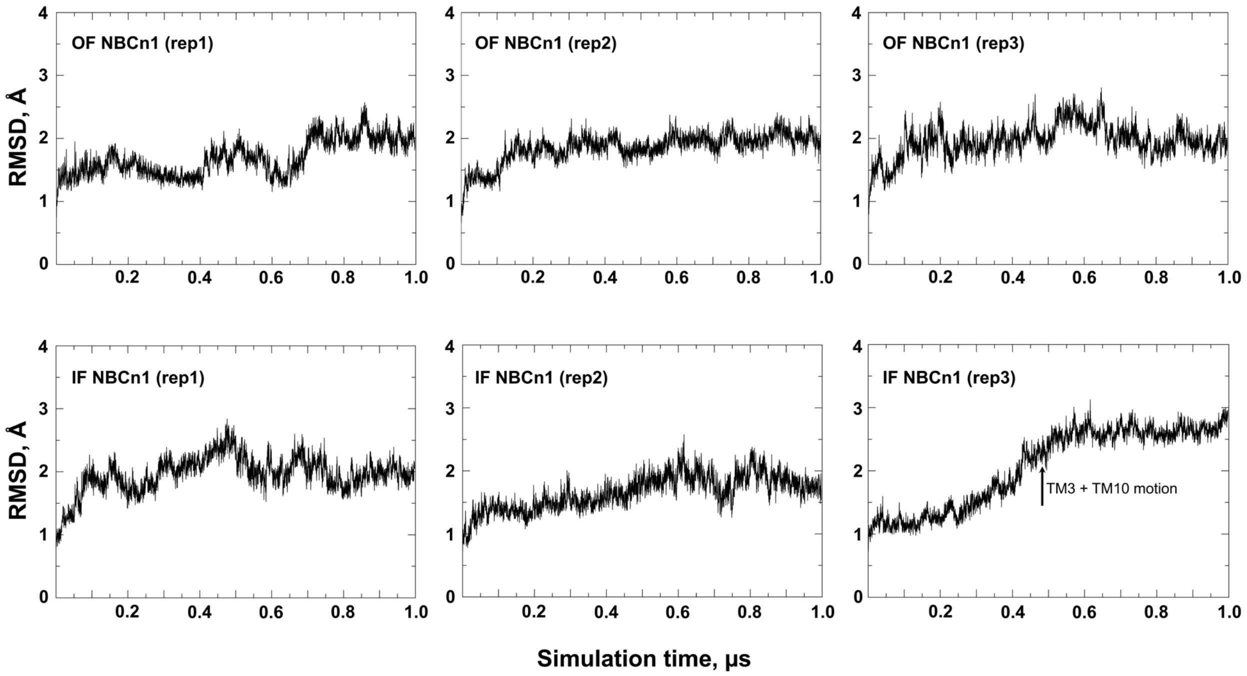

Supplementary Fig. S14. RMSD plots of the unconstrained 1 µs MD simulations with apo-NBCn1 in the OF and IF state. The RMSD values were calculated for the Cα atoms of the transmembrane domain helices with all loops excluded from the calculations due to their high flexibility.

Supplementary Table 1. Data collection, refinement and validation statistics

**Data collection, refinement, and validation statistics**			
**Data collection and Preprocessing**			
Electron microscope	FEI Titan Krios		
Voltage (kV)	300		
Electron detector	Gatan K3 Summit		
Magnification	81,000		
Pixel size (Å)	1.1		
Electron dose (e-/Å^2)	~47		
Defocus range (μm)	-1.8 to -2.4		
CryoSparc	MotionCor, CTF		
**3D reconstruction**			
State	state1	state2	state3
Symmetry imposed	C2	C1	C2
Map resolution (Å)	3.5	3.4	3.3
FSC threshold	0.143	0.143	0.143
Particles for final refinement	114,740	153,169	164,177
**Model building and Refinement**			
Software	COOT, EMBuilder, PHENIX		
Model resolution (Å)	3.8		
FSC threshold	0.5		
Map sharpening B-factor (Å^2)	-143		
Model composition			
Non-hydrogen atoms	9100		
Protein residues	9040		
Ligands	122		
R.m.s. deviations			
Bond lengths (Å)	0.003		
Bonds angle (°)	0.653		
**Validation**			
MolProbity score	1.16		
Clashscore	1.3		
EMRinger score	2.1		
Poor rotamers (%)	0		
Ramachandran plot statistics (%)			
Preferred (%)	93.15		
Allowed (%)	6.5		
Outlier (%)	0.35		

Supplementary Table 2. Absolute free energy of binding (ΔG) calculations

	$G(2Na^+\text{-}CO_3^{2-})$	$\Delta G(2Na^+\text{-}CO_3^{2-})$	$G(Na^+\text{-}HCO_3^-)$	$\Delta G(Na^+\text{-}HCO_3^-)$
Water	-291.91 ± 0.30		-90.37 ± 0.14	
OF State	-364.22 ± 1.57	-72.30 ± 1.57	-93.35 ± 0.78	-2.98 ± 0.78
IF State	-388.72 ± 1.73	-96.81 ± 1.73	-101.31 ± 0.67	-10.94 ± 0.67

Absolute free energy of binding (ΔG) calculations (in kcal/mol) from the last 4 ns of simulations. ΔG(i) are calculated as $\Delta G(i) = G_{state}(i) - G_{water}(i)$. Negative ΔG value corresponds to stabilized ion combination in the $S1^{MD}$ site with respect to the ion combination in a water box. The values in the table represent the mean ± SD of the points obtained in the last 4 ns of each simulation.

Supplementary Table 3. NBCn1 flux, transporter (monomer) number and TOR values

Flux (mol·cell^{-1}·s^{-1})	PM monomers (mol·cell^{-1})	TOR (s^{-1})
8.78 ± 1.15 x 10^{-16}	5.77 ± 0.35 x 10^{-20}	15,211 ± 2,197
n = 9	n = 8	n = 9

PM = plasma membrane

The average cell volume (L) in these experiments was 1.99 ± 0.14 x 10^{-12} (n=35).

Supplementary Table 4. Details of the simulations

System	Calculation type	System composition and size	# replicas	Duration per replica
OF NBCn1	SILCS (MD part)	Variable number of atoms<br>- apo OF NBCn1 monomer<br>- POPC:CHOL = 9:1<br>- 55M H_2O<br>- 8 types of probes (250 mM each)<br>Size along x and y: 120 x 120 Å	10	100 ns
IF NBCn1	SILCS (MD part)	Variable number of atoms<br>- apo OF NBCn1 monomer<br>- POPC:CHOL = 9:1<br>- 55M H_2O<br>- 8 types of probes (250 mM each)<br>Size along x and y: 120 x 120 Å	10	100 ns
OF NBCn1 (+2Na^+-CO_3^{2-})	MD<br>z - position restraints on CO_3^{2-}	- truncated OF NBCn1 monomer bound to 2Na^+-CO_3^{2-}<br>- 211 POPC (109 top, 102 bottom)<br>- 18607 H_2O<br>- 92063 atoms total<br>- 150 mM NaCl<br>Size: 96 x 96 x 108 Å	71	100 ns
IF NBCn1 (+2Na^+-CO_3^{2-})	MD<br>z - position restraints on CO_3^{2-}	- truncated IF NBCn1 monomer bound to 2Na^+-CO_3^{2-}<br>- 211 POPC (109 top, 102 bottom)<br>- 18607 H_2O<br>- 92063 atoms total<br>- 150 mM NaCl<br>Size: 96 x 96 x 108 Å	71	100 ns
OF NBCn1 (+2Na^+-CO_3^{2-})	MD<br>unrestrained	- OF NBCn1 monomer bound to 2Na^+-CO_3^{2-}<br>- 366 POPC (186 top, 180 bottom)<br>- 45919 H_2O<br>- 196581 atoms total<br>- 75 mM NaCl +37.5 mM $NaHCO_3$ + 37.5 mM Na_2CO_3<br>Size: 120 x 120 x 140 Å	3	200 ns
IF NBCn1 (+2Na^+-CO_3^{2-})	MD<br>unrestrained	- IF NBCn1 monomer bound to 2Na^+-CO_3^{2-}<br>- 366 POPC (186 top, 180 bottom)<br>- 43798 H_2O<br>- 189834 atoms total<br>- 75 mM NaCl +37.5 mM $NaHCO_3$ + 37.5 mM Na_2CO_3<br>Size: 120 x 120 x 140 Å	3	200 ns
OF NBCn1	MD<br>unrestrained	- apo truncated OF NBCn1 monomer<br>- 366 POPC (186 top, 180 bottom)<br>- 34877 H_2O<br>- 162227 atoms total<br>- 75 mM NaCl +37.5 mM $NaHCO_3$ + 37.5 mM Na_2CO_3<br>Size: 120 x 120 x 120 Å	3	1000 ns
IF NBCn1	MD<br>unrestrained	- apo truncated IF NBCn1 monomer<br>- 366 POPC (186 top, 180 bottom)	3	1000 ns

		- 30621 H_2O<br>- 149057 atoms total<br>- 75 mM NaCl +37.5 mM $NaHCO_3$ + 37.5 mM Na_2CO_3<br>Size: 120 x 120 x 110 Å		
OF NBCn1 (+$2Na^+$-CO_3^{2-})	FEP/λ-REMD	- truncated OF NBCn1 monomer bound to $2Na^+$-CO_3^{2-}<br>- 211 POPC (109 top, 102 bottom)<br>- 17089 H_2O<br>- 87499 atoms total<br>- 150 mM NaCl<br>Size: 96 x 96 x 103 Å	32	10 ns
OF NBCn1 (+Na^+-HCO_3^-)	FEP/λ-REMD	- truncated OF NBCn1 monomer bound to Na^+-HCO_3^-<br>- 211 POPC (109 top, 102 bottom)<br>- 17089 H_2O<br>- 87500 atoms total<br>- 150 mM NaCl<br>Size: 96 x 96 x 103 Å	32	10 ns
IF NBCn1 (+$2Na^+$-CO_3^{2-})	FEP/λ-REMD	- truncated IF NBCn1 monomer bound to $2Na^+$-CO_3^{2-}<br>- 211 POPC (109 top, 102 bottom)<br>- 18359 H_2O<br>- 91317 atoms total<br>- 150 mM NaCl<br>Size: 96 x 96 x 106 Å	32	10 ns
IF NBCn1 (+Na^+-HCO_3^-)	FEP/λ-REMD	- truncated IF NBCn1 monomer bound to Na^+-HCO_3^-<br>- 211 POPC (109 top, 102 bottom)<br>- 18359 H_2O<br>- 91318 atoms total<br>- 150 mM NaCl<br>Size: 96 x 96 x 106 Å	32	10 ns
$2Na^+$-CO_3^{2-} water box	FEP/λ-REMD	- 2 Na^+<br>- 1 CO_3^{2-}<br>- 4715 H_2O<br>- 14151 atoms total<br>Size: 53 x 53 x 53 Å	32	10 ns
Na^+-HCO_3^- water box	FEP/λ-REMD	- 1 Na^+<br>- 1 HCO_3^-<br>- 4718 H_2O<br>- 14160 atoms total<br>Size: 53 x 53 x 53 Å	32	10 ns
NBCn1 intermediate Climber structures	MD (unrestrained part)	- apo truncated intermediate NBCn1 monomers from Climber<br>- 233 POPC (120 top, 113 bottom)<br>- 20206 H_2O<br>- 99878 atoms total<br>- 75 mM NaCl +37.5 mM $NaHCO_3$ + 37.5 mM Na_2CO_3<br>Size: 100 x 100 x 108 Å	2 x 16	1000 ns

Supplementary References:

1. Kucukelbir, A., Sigworth, F. J. & Tagare, H. D. Quantifying the local resolution of cryo-EM density maps. *Nat. Methods* **11**, 63-65 (2014).
2. Marino, A. A. et al. Association between cell membrane potential and breast cancer. *Tumour Biol.* **15**, 82-89 (1994).
