# Supplementary material for: CryoEM and computational modeling structural insights into the pH regulator NBCn1
Source: Nat Commun. 2025 Nov 11;16:9932. doi: 10.1038/s41467-025-64868-z (PMC12606367; doi:10.1038/s41467-025-64868-z)
Supplement: Supplementary file 2 — Description of Additional Supplementary Files [file 41467_2025_64868_MOESM2_ESM.pdf]

Description of Additional Supplementary Files

Supplementary Data 1: Forward and reverse primers used to generate NBCn1 mutant constructs.

Supplementary Data 2: Initial OF and IF NBCn1 + $2Na^{+}$ + CO_3^{2-} and NBCn1 + Na^{+} + CO_3^{2-} structures for calculations of free energy of ion binding.

Supplementary Data 3: Structures (beads) produced by the Climber simulations of the OF to IF transition in apo-NBCn1.

Supplementary Data 4: First and last step of the 200 ns unrestrained MD trajectories of OF and IF NBCn1 + $2Na^{+}$ + CO_3^{2-} (3 replicas per system).

Supplementary Data 5: First and last step of the 100 ns MD simulation with applied position restraints (movement along z restrained and movement in the xy plane allowed) of the OF and IF NBCn1 + $2Na^{+}$ + CO_3^{2-} (71 simulation windows per system).

Supplementary Data 6: First and last step of the 1μs unrestrained MD simulations, starting from intermediate structures along the OF to IF transition in apo-NBCn1 (2 sets, 16 replicas per set).

Supplementary Data 7: First and last step of the 1μs unrestrained MD Anton2 trajectories of OF and IF apo NBCn1 (3 replicas per system).

Supplementary Movie 1: Elevator-type core domain (orange) motion relative to gait domain (purple) during the OF to IF transition in human NBCn1. The overall downward shift of the core is ~5 Å combined with a small lateral movement in the xy plane. Significant motion of IL5 (cyan) can be seen.
