# Supplementary material for: CryoEM and computational modeling structural insights into the pH regulator NBCn1
Source: Nat Commun. 2025 Nov 11;16:9932. doi: 10.1038/s41467-025-64868-z (PMC12606367; doi:10.1038/s41467-025-64868-z)
Supplement: Supplementary file 11 — Reporting Summary [file 41467_2025_64868_MOESM11_ESM.pdf]

nature portfolio

Corresponding author(s): Ira Kurtz

Last updated by author(s): August 25, 2025

Reporting Summary

Nature Portfolio wishes to improve the reproducibility of the work that we publish. This form provides structure for consistency and transparency in reporting. For further information on Nature Portfolio policies, see our Editorial Policies and the Editorial Policy Checklist.

Statistics

For all statistical analyses, confirm that the following items are present in the figure legend, table legend, main text, or Methods section.

n/a	Confirmed	
☐	☒	The exact sample size (*n*) for each experimental group/condition, given as a discrete number and unit of measurement
☒	☐	A statement on whether measurements were taken from distinct samples or whether the same sample was measured repeatedly
☐	☒	The statistical test(s) used AND whether they are one- or two-sided<br>*Only common tests should be described solely by name; describe more complex techniques in the Methods section.*
☒	☐	A description of all covariates tested
☒	☐	A description of any assumptions or corrections, such as tests of normality and adjustment for multiple comparisons
☐	☒	A full description of the statistical parameters including central tendency (e.g. means) or other basic estimates (e.g. regression coefficient) AND variation (e.g. standard deviation) or associated estimates of uncertainty (e.g. confidence intervals)
☐	☒	For null hypothesis testing, the test statistic (e.g. *F*, *t*, *r*) with confidence intervals, effect sizes, degrees of freedom and *P* value noted<br>*Give P values as exact values whenever suitable.*
☒	☐	For Bayesian analysis, information on the choice of priors and Markov chain Monte Carlo settings
☒	☐	For hierarchical and complex designs, identification of the appropriate level for tests and full reporting of outcomes
☒	☐	Estimates of effect sizes (e.g. Cohen's *d*, Pearson's *r*), indicating how they were calculated

Our web collection on statistics for biologists contains articles on many of the points above.

Software and code

Policy information about availability of computer code

Data collection	Computational modeling was performed with NAMD 2.14 (https://www.ks.uiuc.edu/Research/namd/), SILCS 2023.1.1 (https://silcsbio.com/software/), Gromacs 2022.6 and 2021.4 (https://manual.gromacs.org/documentation/), Climber v1.0.2011 (https://simtk.org/projects/climber), Anton 2 software version 1.59.0c7 (doi: 10.1109/SC.2014.9), CHARMM-GUI server (http://www.charmm-gui.org/), SWISS-MODELl server (https://swissmodel.expasy.org/), SerialEM was used for data collection https://bio3d.colorado.edu/SerialEM/
Data analysis	cryoSparc Live (https://cryosparc.com/live) was used to select micrographs. Relion3.1 (https://relion.readthedocs.io/en/release-3.1/SPA_tutorial/Introduction.html) and cryoSparc (https://guide.cryosparc.com/) were used for data processing.

For manuscripts utilizing custom algorithms or software that are central to the research but not yet described in published literature, software must be made available to editors and reviewers. We strongly encourage code deposition in a community repository (e.g. GitHub). See the Nature Portfolio guidelines for submitting code & software for further information.

Data

Policy information about availability of data

All manuscripts must include a data availability statement. This statement should provide the following information, where applicable:

- Accession codes, unique identifiers, or web links for publicly available datasets
- A description of any restrictions on data availability
- For clinical datasets or third party data, please ensure that the statement adheres to our policy

The final cryo-EM density map of NBCn1 is deposited to the Electron Microscopy DataBank (EMDB) under the accession code EMD-70906 upon acceptance. The

final atomic model is deposited into the Protein Data Bank (PDB) under the accession code 9OVR. The data are provided in the Supplementary Information files. Source data are provided with this paper.

Research involving human participants, their data, or biological material

Policy information about studies with human participants or human data. See also policy information about sex, gender (identity/presentation), and sexual orientation and race, ethnicity and racism.

Reporting on sex and gender	*Use the terms sex (biological attribute) and gender (shaped by social and cultural circumstances) carefully in order to avoid confusing both terms. Indicate if findings apply to only one sex or gender; describe whether sex and gender were considered in study design; whether sex and/or gender was determined based on self-reporting or assigned and methods used.* *Provide in the source data disaggregated sex and gender data, where this information has been collected, and if consent has been obtained for sharing of individual-level data; provide overall numbers in this Reporting Summary. Please state if this information has not been collected.* *Report sex- and gender-based analyses where performed, justify reasons for lack of sex- and gender-based analysis.*
Reporting on race, ethnicity, or other socially relevant groupings	*Please specify the socially constructed or socially relevant categorization variable(s) used in your manuscript and explain why they were used. Please note that such variables should not be used as proxies for other socially constructed/relevant variables (for example, race or ethnicity should not be used as a proxy for socioeconomic status).* *Provide clear definitions of the relevant terms used, how they were provided (by the participants/respondents, the researchers, or third parties), and the method(s) used to classify people into the different categories (e.g. self-report, census or administrative data, social media data, etc.)* *Please provide details about how you controlled for confounding variables in your analyses.*
Population characteristics	*Describe the covariate-relevant population characteristics of the human research participants (e.g. age, genotypic information, past and current diagnosis and treatment categories). If you filled out the behavioural & social sciences study design questions and have nothing to add here, write "See above."*
Recruitment	*Describe how participants were recruited. Outline any potential self-selection bias or other biases that may be present and how these are likely to impact results.*
Ethics oversight	*Identify the organization(s) that approved the study protocol.*

Note that full information on the approval of the study protocol must also be provided in the manuscript.

Field-specific reporting

Please select the one below that is the best fit for your research. If you are not sure, read the appropriate sections before making your selection.

☒ Life sciences ☐ Behavioural & social sciences ☐ Ecological, evolutionary & environmental sciences

For a reference copy of the document with all sections, see nature.com/documents/nr-reporting-summary-flat.pdf

Life sciences study design

All studies must disclose on these points even when the disclosure is negative.

Sample size	Transport studies: The number of experiments is described under Figure Legends. Cryo-EM studies: The number and identity of the particles that went into each refined map were determined via 3D classification, as described under Methods, Image processing.
Data exclusions	Transport studies: No data exclusion was performed. Cryo-EM studies: Micrographs for which motion correction and ctf fitting were applied, were selected manually by discarding apparent bad ones.
Replication	Transport studies: The number of times each construct was studied is described under Figure Legends. Cryo-EM studies: No replication was performed and all analysis algorithms were deterministic.
Randomization	Transport studies: Randomization was not applicable to these studies. Cryo-EM studies: During auto-3D refinement, data were randomly split into 2 groups following the "gold standard" protocol (doi:10.1038/nmeth.2115), which generated half1 and half2 maps to enable resolution estimation through cross-validation.
Blinding	Transport studies: No blinding was done as the data needed to be known for the analysis. Cryo-EM studies: No blinding was performed as the exact identity of the sample was known for the analysis.

Reporting for specific materials, systems and methods

We require information from authors about some types of materials, experimental systems and methods used in many studies. Here, indicate whether each material, system or method listed is relevant to your study. If you are not sure if a list item applies to your research, read the appropriate section before selecting a response.

Materials & experimental systems

n/a	Involved in the study
☐	☒ Antibodies
☐	☒ Eukaryotic cell lines
☒	☐ Palaeontology and archaeology
☒	☐ Animals and other organisms
☒	☐ Clinical data
☒	☐ Dual use research of concern
☒	☐ Plants

Methods

n/a	Involved in the study
☒	☐ ChIP-seq
☒	☐ Flow cytometry
☒	☐ MRI-based neuroimaging

Antibodies

Antibodies used

V5 Tag Monoclonal Antibody (R960-25) from Thermofisher Scientific
https://www.thermofisher.com/antibody/product/V5-Tag-Antibody-clone-SV5-Pk1-Monoclonal/R960-25

Secondary antibody: Peroxidase AffiniPure Donkey Anti-Mouse IgG (H+L) (715-035-150) from Jackson ImmunoResearch
https://www.jacksonimmuno.com/catalog/products/715-035-150

Anti-SLC4A7/NBCn1 antibody (ab82335) from Abcam
https://www.abcam.com/en-us/products/primary-antibodies/slc4a7-nbcn1-antibody-ab82335?srsltid=AfmBOorMN56p0SGXJ83oqNcSg1TI8dgaZL8YJh8rSufC3trrwf21OYoT

Secondary antibody: Peroxidase AffiniPure Mouse Anti-Rabbit IgG (H+L) (211-035-109) from Jackson ImmunoResearch
https://www.jacksonimmuno.com/catalog/products/211-035-109/Mouse-Rabbit-IgG-HL-Horseradish-Peroxidase

Validation

The primary and secondary antibodies were previously validated in numerous studies (see above company websites)

Eukaryotic cell lines

Policy information about cell lines and Sex and Gender in Research

Cell line source(s)

HEK293 human embryonic kidney cell line was purchased from the ATCC: 293 [HEK-293] (ATCC® CRL-1573™).

Authentication

Authentication was performed by ATCC.

Mycoplasma contamination

No mycoplasma contamination purity was stated by the provider.

Commonly misidentified lines
(See ICLAC register)

None

Plants

Seed stocks

Report on the source of all seed stocks or other plant material used. If applicable, state the seed stock centre and catalogue number. If plant specimens were collected from the field, describe the collection location, date and sampling procedures.

Novel plant genotypes

Describe the methods by which all novel plant genotypes were produced. This includes those generated by transgenic approaches, gene editing, chemical/radiation-based mutagenesis and hybridization. For transgenic lines, describe the transformation method, the number of independent lines analyzed and the generation upon which experiments were performed. For gene-edited lines, describe the editor used, the endogenous sequence targeted for editing, the targeting guide RNA sequence (if applicable) and how the editor was applied.

Authentication

Describe any authentication procedures for each seed stock used or novel genotype generated. Describe any experiments used to assess the effect of a mutation and, where applicable, how potential secondary effects (e.g. second site T-DNA insertions, mosiacism, off-target gene editing) were examined.
